# Global behavior of epidemic transmission on heterogeneous networks via two distinct routes

**DOI:** 10.1186/1753-4631-2-2

**Published:** 2008-05-01

**Authors:** Haifeng Zhang, Michael Small, Xinchu Fu

**Affiliations:** 1 School of Mathematics and Computational Science, Anhui University, Hefei 230039, China; 2 Department of Electronic and Information Engineering, Hong Kong Polytechnic University, Hung Hom, Kowloon, Hong Kong, China; 3 Department of Mathematics, Shanghai University, Shanghai 200444, China

## Abstract

In the study of epidemic spreading two natural questions are: whether the spreading of epidemics on heterogenous networks have multiple routes, and whether the spreading of an epidemic is a local or global behavior? In this paper, we answer the above two questions by studying the SIS model on heterogenous networks, and give the global conditions for the endemic state when two distinct routes with uniform rate of infection are considered. The analytical results are also verified by numerical simulations.

## Introduction

The dynamical behaviors of epidemic diseases have been studied for quite a long time, SIS and SIR are the two important and fundamental epidemic models [1]. For the SIS epidemic model, each individual can exist in two states: S-susceptible and I-infected. At each time, the susceptible individual which is connected to an infected neighbor will be infected with rate *λ*. Meanwhile, the infected individuals may be recovered and become susceptible individuals at a rate *γ*. For the SIR model, there are two different aspects from the SIS model: on one hand, individuals can exist in another state: R-recovered/removed; on the other hand, once an infected individual becomes recovered then the individual cannot be infected again.

In order to explore the mechanism of the evolution of complex networks, in 1999, Barabási and Albert addressed a new model of complex networks: scale-free networks (BA) [2]. In a scale-free network the probability *P*(*k*) for any node with *k *links to other nodes is distributed according to the power law *P*(*k*) ~ *k*^-*γ*^. Then researchers found that many real complex systems are scale-free networks, such as the WWW (World Wide Web), the Internet, and so on. These types of networks are considered as heterogenous networks (with a degree distribution exhibiting large fluctuations). Because many epidemic diseases occur in communities exhibiting characteristics consistent with heterogenous networks, many researchers studied the mechanism of the spreading of epidemics on heterogenous networks [3-10] (see also Zhang HF, Fu XC: The spread of epidemics on scale-free networks with nonlinear infectivity. *Nonl Anal, Series A*, in press, and d'Onofrio A: A note on the global behaviour of the network-based SIS epidemic model. *Nonl Anal Real World Appl*, in press).

For the mechanism of the spreading of epidemic on complex networks, different researcher gave different explanations [3,6,10]. For instance, Pastor-Satorras et al. concluded that the epidemic threshold *λ*_*c *_= 0 for heterogenous networks with sufficiently large size [3], and Zhou et al suggested that the threshold *λ*_*c *_is a constant value, regardless of the size of networks and the degree distribution [6]. Both of the results were obtained just by considering one route of spreading epidemic, and the corresponding results are too special to completely reflect the mechanism of spreading of epidemics. Contrary to the above assumptions, many diseases can be spread in many ways, and they have positive thresholds which are relevant to the degree distribution and the size of networks, e.g, people transmit HIV by having unprotected sex, by receiving infected blood transfusions or, through birth. Furthermore, some epidemics just can prevail in a local place, and some kind of epidemics will globally prevail. In order to better explain the mechanism of the spreading of epidemic on complex networks, and answer whether the spreading of an epidemic is a local or global behavior, we consider two distinct routes of spreading of epidemics on heterogenous networks, and obtain some new results which are not given previously.

The rest of this paper is organized as follows: In Section 2, we study two routes of spreading epidemics on complex networks by a method [11] (and see d'Onofrio A: A note on the global behaviour of the network-based SIS epidemic model. *Nonl Anal Real World Appl*, in press) which answers explicitly whether the spreading of an epidemic is a local or global behavior. Though the threshold can also be obtained by solving a self-consistency equation [3-5], however, such method did not answer whether the endemic state is locally or globally stable. In order to demonstrate the advantage of the method we used [11] (also d'Onofrio A: A note on the global behaviour of the network-based SIS epidemic model. *Nonl Anal Real World Appl*, in press), another two routes of spreading epidemics is considered in Section 3, here the threshold cannot be obtained by solving a self-consistency equation. In Section 4, we present numerical simulations to verify our analytical results. And finally, conclusions are given in Section 5.

## When the threshold can be obtained by solving a self-consistency equation

As for the mechanism of the spreading of epidemics on heterogeneous networks, R. Pastor-Satorras et al considered that the infective capability of infected individuals is proportional to their degrees, and the factor $\Theta =\sum_{k^{\prime }=1}^{k_{max}}P(k^{\prime }|k)I_{k^{\prime }}$ (here *P *(*k'*|*k*) denotes the conditional probability that a node with degree *k *is connected to a node with degree *k'*, *k*_*max *_is the maximal degree of the networks, and *I*_*k' *_is the density of infected individuals with degree *k'*) was used to stand for the probability that an edge emanating from a node of degree *k *points to an infected nodes. As a result, the epidemic threshold *λ*_*c *_= 0 for heterogenous networks with sufficiently large size [3-5]. However, in [6], Zhou et al argue that Θ may have another form, they let $\Theta =\sum_{k^{\prime }=1}^{k_{max}}\frac{UP(k^{\prime }|k)I_{k^{\prime }}}{k^{\prime }}$, just because every infected individual may have the same infective capability *U *to infect other individuals. And they obtained the epidemic threshold $\lambda_{c}=\frac{1}{U}$, no matter the individuals' degree and the size of scale-free networks.

Just as we mentioned in above section, both of them have limitations, so we consider that both of these mechanisms are concurrent on complex networks.

For the heterogeneous networks, we also classify individuals into groups according to their degrees, then *S*_*k *_and *I*_*k *_are the density of susceptible individuals and the density of infected individuals with degree *k *respectively, so *S*_*k *_+ *I*_*k *_= 1.

Now we give the dynamical equations for the epidemic on complex networks:

(1) $$\begin{array}{ccc} \frac{dI_{k}(t)}{dt} & = & -I_{k}+p\lambda k(1-I_{k})\Theta_{1}+\ldots \\ & & (1-p\beta (1-I_{k})k\Theta_{2}, \end{array}$$

for *k *= 1, 2, ..., *k*_*max *_and where *λ*, *β *are the two different infective rates, and the recovery rate is assumed to be unity, the parameter *p*, (0 ≤ *p *≤ 1) gives the different ratio between two routes of spreading epidemic. We suppose the degree distribution is uncorrelated, that is, $P(k^{\prime }|k)=\frac{k^{\prime }P(k^{\prime })}{\langle k\rangle }$, then we have

(2) $$\begin{array}{c} \Theta_{1}=\sum_{k^{\prime }=1}^{k_{max}}\frac{k^{\prime }P(k^{\prime })I_{k^{\prime }}}{\langle k\rangle }\\ \Theta_{2}=\sum_{k^{\prime }=1}^{k_{max}}\frac{UP(k^{\prime })I_{k^{\prime }}}{\langle k\rangle } \end{array}$$

where $\langle k\rangle =\sum_{k^{\prime }=1}^{k_{max}}k^{\prime }P(k^{\prime })$. Then Eq(1) can be written in a compact form:

(3) $$\begin{array}{lll} \frac{dI_{k}(t)}{dt} & = & -I_{k}+\ldots \\ & & (1-I_{k})k\sum_{k^{\prime }=1}^{k_{max}}\frac{(p\lambda k^{\prime }+(1-p)U\beta )P(k^{\prime })I_{k^{\prime }}}{\langle k\rangle } \end{array}$$

(where *k *= 1, 2, ..., *k*_*max*_). By letting *I *= [*I*_1_, *I*_2_, ..., $I={\left[I_{1},I_{2},\cdots ,I_{k_{max}}\right]}^{T}$, Eq(3) can be rewritten as a vector form:

(4) $$\frac{dI(t)}{dt}=AI+N(I,t)$$

where *AI *is the linear part of *I*, and *N*(*I*, *t*) is the nonlinear part of *I*, and

(5) $$A_{kk^{\prime }}=-\delta_{kk^{\prime }}+\frac{k(p\lambda k^{\prime }+U(1-p)\beta )P(k^{\prime })}{\langle k\rangle }$$

(*k*, *k' *= 1, 2, ..., *k*_*max*_) where *δ*_*kk' *_= 1 if *k *= *k'*, or *δ*_*kk' *_= 0 otherwise.

(6) $$\begin{array}{lll} N_{k} & = & -I_{k}k\sum_{k^{\prime }=1}^{k_{max}}\frac{(p\lambda k^{\prime }+U(1-p)\beta )P(k^{\prime })I_{k^{\prime }}}{\langle .k\rangle }\\ & < & 0 \end{array}$$

(*k *= 1, 2, ..., *k*_*max*_). By some computation, we can find that, for the matrix *A*, there are (*k*_*max *_- 1) eigenvalues equal to -1.

In order to find the last eigenvalue of *A*, we let

(7) *V *= [*λ*, 2*λ*, ..., N*λ*]^*T*^

and write matrix *A *as

(8) $$\begin{array}{lll} A & = & -E_{k_{max}}+\ldots \\ & & \frac{1}{\langle k\rangle }{\left[\begin{array}{c} (p\lambda +U(1-p)\beta )P(1)V\\ (2p\lambda +U(1-p)\beta )P(2)V\\ \vdots \\ (Np\lambda +U(1-p)\beta )P(N)V \end{array}\right]}^{T} \end{array}$$

where $E_{k_{max}}$ is an identity matrix, then we have

(9) $$\begin{array}{lll} AV & = & (-1+\ldots \\ & & \frac{1}{\langle k\rangle }\sum_{k^{\prime }=1}^{k_{max}}({k^{\prime }}^{2}p\lambda +Uk^{\prime }(1-p)\beta )P(k^{\prime }))V\\ & = & (-1+p\lambda \frac{\langle k^{2}\rangle }{\langle k\rangle }+(1-p)U\beta )V \end{array}$$

From Eq (9), it follows that the *k*_*max*_'s eigenvalue of the matrix *A *is

(10) $$u=-1+p\lambda \frac{\langle k^{2}\rangle }{\langle k\rangle }+(1-p)U\beta$$

If the solution *I *= **0 **of the Eq(3) is stable, all eigenvalues of the matrix *A *must be non-positive, that is, *u *≤ 0.

So we have the following conclusion:

If

(11) $$p\lambda \frac{\langle k^{2}\rangle }{\langle k\rangle }+(1-p)U\beta \leq 1$$

*then the solution I = **0 **of the Eq(3) is globally asymptotically stable, otherwise the unique endemic solution $I=[I_{1},I_{2},...,I_{k_{max}}]>0$ (**0 **is a zero vector) is globally asymptotically stable, except when there are no infected individuals in the networks at the initial time*.

From the inequality (11), we can find the thresholds for the outbreak of epidemics on complex networks with the two routes of spreading epidemic mentioned above, and at the same time we can answer whether the endemic state is globally stable.

When *p *= 1, that is, there is only one way of spreading of epidemic on complex networks, as discussed in [3], then we obtain the threshold for $\lambda >\frac{\langle k\rangle }{\langle k^{2}\rangle }$, the same as the result given in [3-5].

When *p *= 0, then we obtain the threshold for $\beta >\frac{1}{U}$, the same as the result given in [6].

When 0 <*p *< 1, i.e., there are two routes of spreading of epidemic on complex networks, our result suggest that the threshold for the outbreak of epidemic is positive, which is relevant to the ratio of two routes of spreading of epidemic, the degree distribution, and the size of the networks. So the threshold we obtain is neither zero nor just a constant given by previous authors.

## When threshold cannot be obtained by solving a self-consistency equation

In the above section, the threshold for the case we considered can also be obtained by solving a self-consistency equation, although this does not explicitly determine whether the equilibrium is globally stable. In this section, in order to demonstrate the advantage of the method we used in the above section, we consider another case where the threshold cannot be obtained by solving a self-consistency equation. Moreover, such case may exist in the real networks.

We suppose that there are also two routes of the spreading epidemics on complex networks, one route is given by Θ_1_, another route of the spreading of epidemic is the standard SIS model [1].

The dynamical equations are

(12) $$\begin{array}{lll} \frac{dI_{k}(t)}{dt} & = & -I_{k}+\ldots \\ & & \lambda p(1-I_{k})\frac{k}{\langle k\rangle }\sum_{k^{\prime }=1}^{k_{max}}k^{\prime }P(k^{\prime })I_{k^{\prime }}+\ldots \\ & & (1-I_{k})(1-p)\beta (k)\sum_{k^{\prime }=1}^{k_{max}}P(k^{\prime })I_{k^{\prime }} \end{array}$$

(*k *= 1, ..., *k*_*max*_). Here, we should distinguish the second term of the right-hand side of Eq(12) from the second term of the right-hand side of Eq(3). In Eq(3), Zhou et al considered that the susceptible individuals which may be infected are proportional to their degrees *k *though they are irrelevant to the infected individuals, however, in Eq(12) we suppose that all of the susceptible individuals which may be infected are proportional to a constant rate, i.e. (*k*).

By using the same method as in Section 2, we can rewrite Eq(12) in the following vector form,

(13) $$\frac{dI(t)}{dt}=AI+N(I,t)$$

and

(14) $$A_{kk^{\prime }}=-\delta_{kk^{\prime }}+\frac{kp\lambda k^{\prime }P(k^{\prime })}{\langle k\rangle }+\langle k\rangle (1-p)\beta P(k^{\prime })$$

(15) $$\begin{array}{lll} N_{k} & = & -I_{k}\sum_{k^{\prime }=1}^{k_{max}}[\frac{kp\lambda k^{\prime }}{\langle k\rangle }+\langle k\rangle (1-p)\beta ]P(k^{\prime })I_{k^{\prime }}\\ & < & 0 \end{array}$$

(*k*, *k' *= 1, 2, ..., *k*_*max*_).

By solving the matrix *A*, we can find that there are *k*_*max *_- 2 eigenvalues equal to -1, and the other two eigenvalues *u *are given by the following equation

(16) $$\begin{array}{c} u^{2}-[\lambda p+(1-p)\beta \langle k\rangle +\frac{\lambda p}{\langle k\rangle }T_{1}-2]u+\ldots \\ \{(1-p)p\beta \lambda T_{1}-\lambda p-(1-p)\beta \langle k\rangle -\ldots \\ \frac{\lambda p}{\langle k\rangle }T_{1}+1-(1-p)p\lambda \beta \langle k\rangle T_{2}\}=0 \end{array}$$

where

(17) $$\begin{array}{ccc} T_{1} & = & \sum_{k^{\prime }=2}^{k_{max}}k^{\prime }(1-k^{\prime })P(k^{\prime })\\ & = & \langle k^{2}\rangle -\langle k\rangle .\\ T_{2} & = & \sum_{k^{\prime }=2}^{k_{max}}\left(1-k^{\prime })P(k^{\prime }\right)\\ & = & \langle k\rangle -1 \end{array}$$

From Eq(16), (17), we have

(18) $$\begin{array}{c} u^{2}-[(1-p)\beta \langle k\rangle +\frac{\lambda p\langle k^{2}\rangle }{\langle k\rangle }-2]u+\ldots \\ \{(1-p)p\beta \lambda \langle k^{2}\rangle -(1-p)\beta \langle k\rangle -\ldots \\ \frac{\lambda p\langle k^{2}\rangle }{\langle k\rangle }+1-(1-p)p\lambda \beta {\langle k\rangle }^{2}\}=0 \end{array}$$

In order to obtain both of the negative eigenvalues, by solving Eq(18) we have

If

(19) $$\begin{array}{c} 1\geq \frac{1}{2}[\frac{\lambda p\langle k^{2}\rangle }{\langle k\rangle }+(1-p)\beta \langle k\rangle ]+\ldots \\ \;\;\frac{1}{2}{\left\{{\left[\frac{\lambda p\langle k^{2}\rangle }{\langle k\rangle }-(1-p)\beta \langle k\rangle \right]}^{2}+4(1-p)p\lambda \beta {\langle k\rangle }^{2}\right\}}^{1/2} \end{array}$$

*then the solution I *= ***0 ****of the Eq(15) is globally asymptotically stable, otherwise the unique endemic solution *$I=[I_{1},I_{2},\cdots ,I_{k_{max}}]\neq 0$*(**0 **is a zero vector) is globally asymptotically stable. Except when there are no infected individuals in the networks at the initial time*.

When *p *= 1, then we obtain the threshold for $\lambda >\frac{\langle k\rangle }{\langle k^{2}\rangle }$, the same as the result given in [3-5]. When *p *= 0, then we obtain the threshold for $\beta >\frac{1}{\langle k\rangle }$, the same as the result on homogenous networks.

If 0 <*p *< 1, we can find the threshold for this two routes of spreading epidemic is not just the linear combination of threshold *λ *and *β *as shown in inequality (11), which manifest that some complex behaviors may come forth.

We remark here that we can also consider more routes' spreading of epidemics on complex networks, by using the similar method, although here in this paper we have only considered two routes of spreading epidemics.

## Numerical simulations

In this section, we present numerical simulations to support the results obtained in previous sections. Our simulations are based on the BA network with *P*(*k*) = *k*^-*γ*^, *γ *= 3, *N *= 2000, and ⟨*k*⟩ = 6, ⟨*k*^2^⟩ = 98.

**Figure 1:**
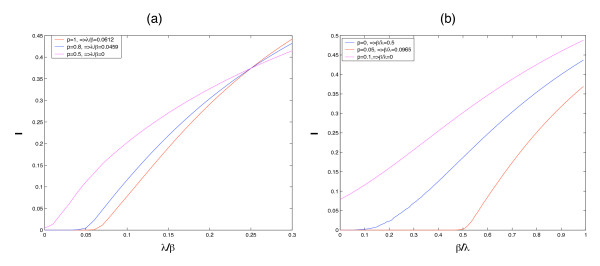
**Verification of inequalities (20) and (21)**. Figure 1 (a) Simulations show that *λ*/*β *is in accordance with inequality (20) by changing *p*. Figure 1 (b) Simulations show that *β*/*λ *is in accordance with inequality (21) by changing *p*.

In Figure 1, our simulations verify the inequality(11). In order to simultaneously consider two infective rates *λ*, *β*, in Figure 1(a), we study the ratio of *λ*/*β *with *p *changing, then inequality (11) can be written as

(20) $$p(\frac{\lambda }{\beta })\frac{\langle k^{2}\rangle }{\langle k\rangle }+(1-p)U\leq 1$$

In Figure 1(b), we study the ratio of *β*/*λ*, with *p *changing, then inequality (11) can be written as

(21) $$p\frac{\langle k^{2}\rangle }{\langle k\rangle }+(1-p)U(\frac{\beta }{\lambda })\leq 1$$

**Figure 2:**
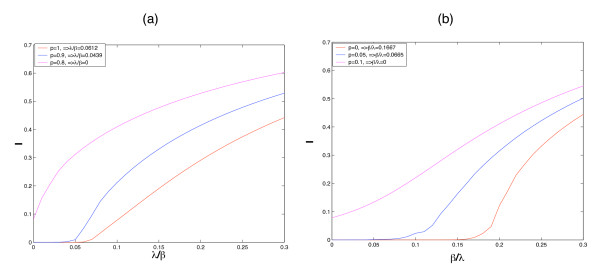
**Verification of inequalities (22) and (23)**. Figure 2 (a) Simulations show that *λ*/*β *is in accordance with inequality (22) by changing *p*. Figure 2 (b) Simulations show that *β*/*λ *is in accordance with inequality (23) by changing *p*.

In Figure 2, our simulations verify the inequality (19). Just as in Figure 1, we simultaneously consider two infective rates *λ*, *β*, in Figure 2(a), we study the ratio of *λ*/*β *with *p *changing, then inequality (19) can be written as

(22) $$\begin{array}{c} 1\geq \frac{1}{2}[\frac{\lambda }{\beta }\frac{p\langle k^{2}\rangle }{\langle k\rangle }+(1-p)\langle k\rangle ]+\ldots \\ \;\;\frac{1}{2}{\left\{{\left[\frac{\lambda }{\beta }\frac{p\langle k^{2}\rangle }{\langle k\rangle }-(1-p)\langle k\rangle \right]}^{2}+\frac{\lambda }{\beta }4(1-p)p{\langle k\rangle }^{2}\right\}}^{1/2} \end{array}$$

In Figure 2(b), we study the ratio of *β*/*λ*, with p changing, then inequality (19) can be written as

(23) $$\begin{array}{c} 1\geq \frac{1}{2}[\frac{p\langle k^{2}\rangle }{\langle k\rangle }+\frac{\beta }{\lambda }(1-p)\langle k\rangle ]+\ldots \\ \;\;\frac{1}{2}{\left\{{\left[\frac{p\langle k^{2}\rangle }{\langle k\rangle }-\frac{\beta }{\lambda }(1-p)\langle k\rangle \right]}^{2}+\frac{\beta }{\lambda }4(1-p)p{\langle k\rangle }^{2}\right\}}^{1/2} \end{array}$$

**Figure 3:**
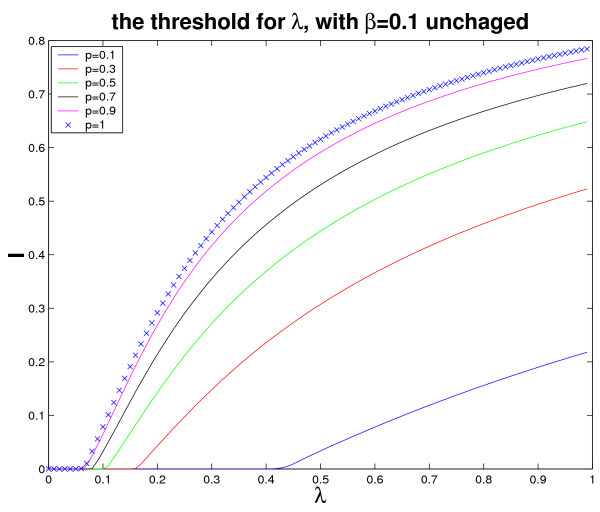
**Verification of inequality (19)**. Simulations show that *λ *is in accordance with inequality (19) by changing *p*. And we can see that the smaller *p *is, the larger the threshold for *λ *is.

**Remark**: *From the above figures, we can find that the thresholds are very small, this is because that we are simulating the ratios between two parameters λ, β. In fact, if we fix one of them, and simulate the threshold for another parameter by changing p, here p can go from *0 *to *1. *For instance, in order to demonstrate this case, we give simulations to verify inequality (19), by letting β *= 0.1 *unchanged, and consider the threshold for λ, with p changing (see Figure *3).

## Conclusion

In order to explain the mechanism of the spreading of epidemic, many results were proposed by researchers. These results were often based on that the spreading of epidemics on complex networks via a single mechanism, hence, these results were often not so realistic. Moreover, many results just gave the threshold for the outbreak of epidemics, which can not explicitly answer whether the epidemic will prevail locally or globally.

Because many diseases can spread in different ways, e.g., HIV virus, the avian flu, and so on, in this paper we consider that the spreading of epidemics on complex networks via multiple routes. Under such assumption, we get positive thresholds, neither a constant value nor a zero value, which are more realistic. Furthermore, we answer that the epidemic will prevail globally once some conditions are satisfied.

In Section 3, the results can be obtained by solving a self-consistency equation, and the threshold is the linear combination of *λ *and *β*. However, when we consider the case as in Section 4, the results can not be obtained by solving a self-consistency equation, moreover, the threshold is not the linear combination of *λ *and *β*, which suggest that the threshold for the multiple routes of spreading epidemic is not just the addition of the respective way of spreading epidemic, sometimes, some complex behavior can happen. Finally, in Section 4, we present numerical simulations to support our analytical results.

## Authors' contributions

All authors contributed equally to this work.

## References

[B1] Kermack WO, McKendrick AG (1927). Contributions to the mathematical theory of epidemics. Proc Roy Soc.

[B2] Barabási AL, Albert R (1999). Emergence of scaling in random networks. Science.

[B3] Pastor-Satorras R, Vespignani A (2001). Epidemic dynamics and endemic states in complex networks. Phys Rev E Stat Nonlin Soft Matter Phys.

[B4] Pastor-Satorras R, Vespignani A (2001). Epidemic spreading in scale-free networks. Phys Rev Lett.

[B5] Pastor-Satorras R, Vespignani A (2002). Infection dynamics in finite size scale-free networks. Phys Rev.

[B6] Zhou LJ, Gea T (2006). Behaviors of susceptible-infected epidemics on scale-free networks with identical infectivity. Phys Rev E Stat Nonlin Soft Matter Phys.

[B7] Small M, Tse CK (2005). Small world and scale free model of transmission of SARS. Int J Bifurcat and Chaos.

[B8] Small M, Tse CK (2005). Clustering model for tranmsmission of the SARS virus: application to epidemic control and risk assesment. Physica.

[B9] Masuda N, Konno N (2006). Multi-state epidemic processes on complex networks. J of Theoretical Biology.

[B10] Fu XC, Small M, Walker DM, Zhang HF (2008). Epidemic dynamics on scale-free networks with piecewise linear infectivity and immunization. Phys Rev.

[B11] Lajmainovitch A, Yorke JA (1976). A deterministic model for gonorrhea in a nonhomogenous population. Math Biosci.

